# The Nature of Impulsivity: Visual Exposure to Natural Environments Decreases Impulsive Decision-Making in a Delay Discounting Task

**DOI:** 10.1371/journal.pone.0097915

**Published:** 2014-05-19

**Authors:** Meredith S. Berry, Mary M. Sweeney, Justice Morath, Amy L. Odum, Kerry E. Jordan

**Affiliations:** Department of Psychology, Utah State University, Logan, Utah, United States of America; Saarland University, Germany

## Abstract

The benefits of visual exposure to natural environments for human well-being in areas of stress reduction, mood improvement, and attention restoration are well documented, but the effects of natural environments on impulsive decision-making remain unknown. Impulsive decision-making in delay discounting offers generality, predictive validity, and insight into decision-making related to unhealthy behaviors. The present experiment evaluated differences in such decision-making in humans experiencing visual exposure to one of the following conditions: natural (e.g., mountains), built (e.g., buildings), or control (e.g., triangles) using a delay discounting task that required participants to choose between immediate and delayed hypothetical monetary outcomes. Participants viewed the images before and during the delay discounting task. Participants were less impulsive in the condition providing visual exposure to natural scenes compared to built and geometric scenes. Results suggest that exposure to natural environments results in decreased impulsive decision-making relative to built environments.

## Introduction

The natural world has long been the subject of human enjoyment and fascination [1], [2], and is often depicted in philosophical writings as healing and rejuvenating [3]. Adults also prefer viewing scenes of the natural world, such as mountains or forests, over human-made environments [4]. Beyond mere preference, exposure to natural environments decreases stress [5], [6], increases happiness [7], improves mood [8], [9], and restores attention [10]. These benefits have led some researchers to characterize exposure to nature as ‘the therapy with no side effects’ [11]. Natural environments rich in biodiversity are also vital to our physical health for medicines, medical research, combating infectious diseases, and food production [12].

Despite the known health and cognitive benefits of interacting with or viewing scenes of nature, it remains unknown whether natural environments may also promote healthy human decision-making. Developing techniques that decrease impulsive, maladaptive human decision-making could promote human and ecological health, as many grave societal and environmental (e.g., climate change) issues can be partially attributed to impulsive human decisions [12], [13]. Impulsivity is a multi-faceted construct that encompasses a number of meanings and can be measured in different ways [14].

The complexity of ‘impulsivity’ is highlighted by the various uses of the term including the failure to wait, inability to withhold a response, and lack of sensitivity to negative or delayed consequences, all of which likely represent different underlying processes [15]. For example, within self-reports, impulsivity manifests across one or more behaviors and personality traits including sensation seeking, distractibility/urgency and behavioral (dys)control [16]. Each of these personality traits can contribute uniquely to ‘impulsivity’ [16]. The focus of the present experiment was to better understand how impulsive decision-making within delay discounting (described below) may be affected by exposure to natural scenes. For this reason, we use delay discounting as a framework to develop the concept of impulsivity.

Impulsivity in many contexts refers to the inability to delay gratification [17], [18], and is associated with the choice of a smaller immediate reward over a larger delayed reward (e.g., the choice to continue eating high fat foods now over a healthier body in the future). One way in which the choice of a smaller immediate reward versus a larger delayed reward can be measured is by delay discounting. Delay discounting refers to the decline of the value of a reward (e.g., money) with the increased time to its receipt [18].

Odum [13], [19] proposed that degree of delay discounting may be a personality trait that is relatively stable across time and contexts. For example, test-retest reliability is good for degree of delay discounting across time and alternate and same versions of the discounting task [20]–[25]. Those who discount money steeply also tend to discount other commodities steeply [13], and degree of delay discounting is similar across real and hypothetical rewards [26]. In other words, an individual who is considered ‘impulsive’ will likely be impulsive across various situations (i.e., impulsivity is not limited to one context or situation). This does not mean, however, that degree of delay discounting is unalterable.

Just as other personality characteristics change with time [27], so does degree of delay discounting as part of normal development processes (i.e., older adults are less impulsive in delay discounting tasks relative to younger adults) [28]. Beyond the change in degree of delay discounting observed as a result of natural development, several training techniques also reduce degree of delay discounting. Some techniques are explicitly designed to decrease impulsive decision-making through a fading procedure, which gradually reduces the delay to the smaller sooner reward, when delays to the smaller sooner and larger later reward were initially equal. These fading procedures have been successful in reducing impulsive decision-making across human and nonhuman populations, and when follow-up measures of delay discounting were assessed, reduced impulsive decision-making remained in the experimental group [29]–[39].

Other techniques have been shown to reduce delay discounting, despite no direct manipulation of the delay discounting task itself [40]. For example, Bickel and colleagues [41] showed working memory training reduced degree of delay discounting. Similarly, financial management training has been associated with decreased impulsive decision-making in a delay discounting task relative to the control group. Importantly, this same financial management training was associated with decreased impulsive decision-making in real world situations unrelated to money [42]. For these reasons, generating general techniques to reduce impulsive decision-making in a delay discounting task may be a useful avenue for future research, and is the focus of the present experiment.

Delay discounting is influenced by both genetic [43] and environmental [44] factors. For example, the heritability of degree of delay discounting has been estimated at up to 50% [43], and the context in which the task is administered can influence degree of discounting [44]. Delay discounting also offers predictive validity and generality across domains and populations [13], [19]. Mitigating impulsive decision-making in delay discounting, therefore, may have broad implications for promoting healthy human choices.

Delay discounting refers to the decrease in subjective value of a delayed reward [18] and is assessed by calculating indifference points: the point at which the value of an immediate outcome is equal to the value of a delayed outcome (e.g., $80 now versus $100 in a year). The more steeply indifference points decline with delay, the more impulsive the decision-making. Delay discounting is well described by a simple hyperbola [18]:
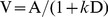
(1)where V is the subjective value of the indifference point, A is the amount of the delayed reward, D is the delay to receipt of the reward, and *k* is the degree to which the value of the reward decreases with delay. The values of A and D are predetermined based on the values used within the research context. For example, if the delayed reward used is 100 dollars, the numerator would be 100. The value of D would be the delay at which the indifference point is generated. Once indifference points are generated at each delay, across a range of delays (e.g., one day to 25 years), then Equation 1 is fit to the indifference points using nonlinear regression, and resulting *k* parameter values (degree of discounting) are compared. The degree of discounting then serves as a comparison across groups or individuals, and offers a measure of impulsive decision-making.

If exposure to natural environments reduces impulsive decision-making (reflected with smaller *k* values), this would empirically support the development of future research endeavors that investigate the potential benefits of nature-based interventions for impulsive decision-making. The present experiment was therefore designed to determine whether exposure to natural environments results in decreased impulsive decision-making relative to built environments. Using a titrating hypothetical monetary discounting task [45], [46] in which indifference points were determined across a range of delays, we tested the effects of viewing photographs of natural or built environments or geometric shapes on impulsive decision-making.

Geometric shapes were chosen as a control condition, allowing for assessment of potential differences in impulsive decision-making as a function of viewing effort [10]. Scenes that require minimal effort to view can restore attention following mentally fatiguing tasks [10]. Kaplan [47] has hypothesized that natural environments require very little effort to view, while human-made environments require more effort to view (and are thus ‘restorative’ versus ‘nonrestorative’, respectively). This is because natural environments do not require directed attention, while built environments do [47]. Differences in eye movements (e.g., saccades, fixations) occurring while viewing natural relative to built environments supports Kaplan’s depiction of less effortful viewing of natural scenes [48]. Geometric shapes, like natural scenes, also require minimal effort to view [10]. Geometric shapes, however, do not depict environments in which humans spend time and are thus considered non-natural. Because natural environments and geometric shapes require little viewing effort [10], these comparisons allow for assessment of potential differences generated across effortless natural and effortless non-natural stimuli.

Previous studies have shown that exposure to scenes of the natural world influences mood, attention and time perception. Specifically, mood is enhanced, attention is restored, and the perception of time slows with viewing scenes of natural environments [8]–[10], [49]. Degree of delay discounting can also be influenced by mood [50], attention [51], and time perception [14]. Some positive mood induction techniques increase impulsive decision-making in delay discounting. Increased attention, as well as slowed time-perception, however, decrease impulsive decision-making in delay discounting. Considering these effects of viewing natural scenes on mood, attention, and time-perception–and the influence that these same cognitive processes exert on degree of delay discounting–we predicted that viewing scenes of nature would result in decreased impulsive decision-making in a delay discounting task relative to viewing scenes of built environments or geometric shapes.

## Method

### Participants

#### Ethics Statement

Undergraduate students (N = 204) were recruited from an introductory psychology course. Participants provided their written informed consent and received course credit for participation. The Utah State University Institutional Review Board approved all experimental procedures.

### Setting and Apparatus

Participants were tested individually in a room equipped with a computer. Experimental manipulations and data recording were programmed using E-Prime 2.0.

### Stimuli

**Figure 1:**
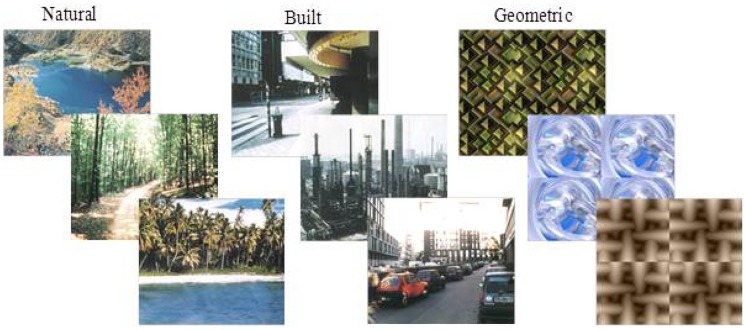
Examples of the stimuli used in natural, built, and geometric conditions. Examples of the stimuli used in the natural (left stimuli), built (center stimuli) and geometric (right stimuli) conditions.

The picture sets used as stimuli have been used in previous experiments testing attention restoration across environments [10]. In the natural condition, participants viewed photographs of natural environments (e.g., forests). In the built condition, participants viewed photographs of human-made environments (e.g., buildings). In the geometric condition (control), participants viewed photographs of geometric shapes (e.g., triangles). There were 25 photographs in each picture set. See Figure 1 for examples of the stimuli used in each condition.

### Procedure

The participant was first seated at the computer and given instructions to choose whichever option they preferred in the delay discounting task [46]. The same instructions were provided on the computer screen to lead the participant through the task. Participants used the mouse to progress through instructional screens and make their choices.

Participants were randomly assigned to one of three conditions–natural (n = 63), built (n = 59), or geometric (n = 63)–using block randomization. Following practice trials, participants viewed condition-specific photographs for 10 s each on the computer screen both prior to the experimental delay discounting task (25 photographs) and between each delay block (5 photographs, selected randomly from the original set of 25 photographs). Excluding photograph type, all aspects of the experiment were identical across conditions.

Participants were tested in the delay discounting task using hypothetical monetary outcomes. All choice screens presented the wording “Would you rather have [amount] now or [amount] in [delay]?” Participants selected either the immediate or delayed outcome to progress; the side of immediate or delayed amount varied randomly across trials. Before experiencing the condition-specific stimuli and completing the titrating amount procedure described below [45], [46], participants completed 10 practice trials. The practice trials were designed to introduce the participant to the interface and choice tradeoffs between amount and delay. Practice trials began with a choice of $10 now or $100 dollars in one week. The immediate option increased by $10 until the final option was a choice between $100 now or $100 in one week. Because practice trials did not titrate based on participant response, they are not included in the delay discounting analysis [46]. Following the 10 practice trials and stimuli exposure, all participants experienced the titrating amount discounting procedure. Delays tested were 1 day, 1 week, 1 month, 6 months, 1 year, 5 years, and 25 years, in that order.

**Figure 2:**
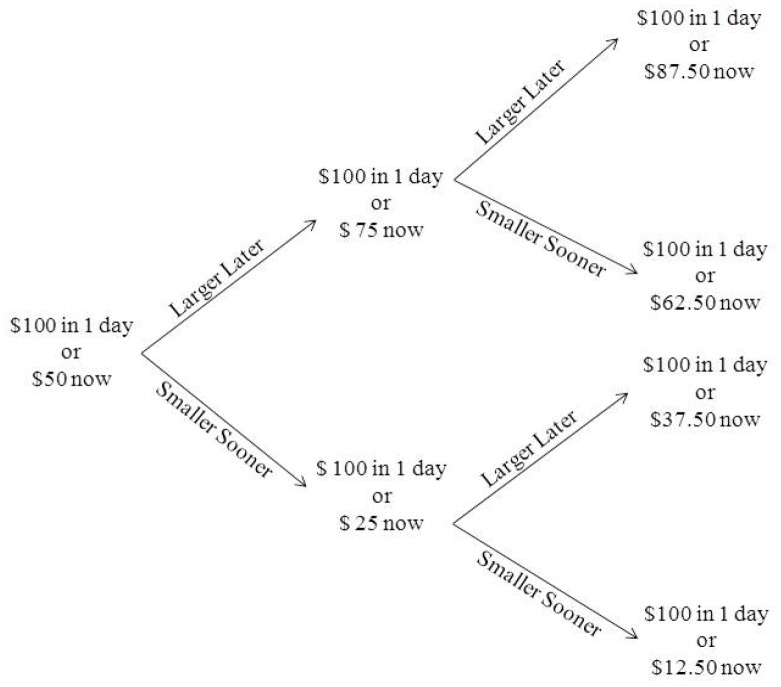
Schematic diagram of potential progression of first three trials of choice procedure. Schematic diagram of the possible outcomes based on participant choice of the smaller sooner or larger later reward for the first three trials of the delay discounting procedure.

For each trial in the titrating amount delay discounting procedure, participants chose between immediate and delayed options. The first trial at each delay began with the choice of $50 now or $100 after a delay, and the immediate amount increased or decreased based on the participant’s response with each subsequent trial. If the immediate outcome was selected, the amount of the next immediate outcome decreased; if the delayed outcome was selected, the amount of the next immediate outcome increased. The adjustment on the first trial was half of the difference between the immediate and delayed outcomes (i.e., $25); for each subsequent trial, the adjustment was half the previous adjustment. For example, if the participant selected the delayed option on the first two trials, the third trial would be $100 delayed option versus $87.50 immediate option. If the participant selected the immediate option on the first two trials, the third trial would be $100 delayed option versus $12.50 immediate option. There were 10 trials at each delay. The indifference point was the last value of the immediate outcome for each delay. See Figure 2 for a schematic diagram of the choice portion of the experiment.

### Data Analysis

Of 204 individuals that participated, data for 19 were not considered due to nonsystematic discounting, which is similar to previous percentages eliminated based on these criteria [52]. Delay discounting data were considered systematic and used if a.) participants discounted more than $5 across any delay (which assumes delay decreases the value of a reward), and b.) indifference points did not increase across consecutive delays by more than 35% of the larger later reward. Substantial increases in the value of a reward across delays suggests that the value of a reward is enhanced with increased delay. These criteria are based on the expectation of a monotonically decreasing discounting function, and are similar to the algorithm used by Johnson and Bickel [52].

Equation 1 [18] was fit to the median indifference points for each condition using nonlinear regression (GraphPad Prism). Equation 1 was also fit to individual indifference points and the resulting *k* values were analyzed to compare degree of discounting across conditions. To determine if there was an overall effect of condition on *k* value (degree of discounting), a Kruskal-Wallis one way analysis of variance (ANOVA) was conducted. This analysis was used because values for *k* are not normally distributed, and require nonparametric statistics [53]. To further compare *k* values, follow-up Mann-Whitney independent two-tailed *t*-tests (used for non-normal distributions like that of *k*) were performed to assess the impact of natural versus built, natural versus geometric, and geometric versus built conditions on *k* values. Effect sizes for non-normal distributions (Cliff’s Delta) were also calculated to compare *k* values across conditions [54]. No other dependent measures were collected or conditions arranged in this study.

## Results

Of the total participants, forty-two percent were males, and two percent chose not to respond to the demographic questions. The mean age was 20.88 (SD = 4.59) years old.

### k Values

**Figure 3:**
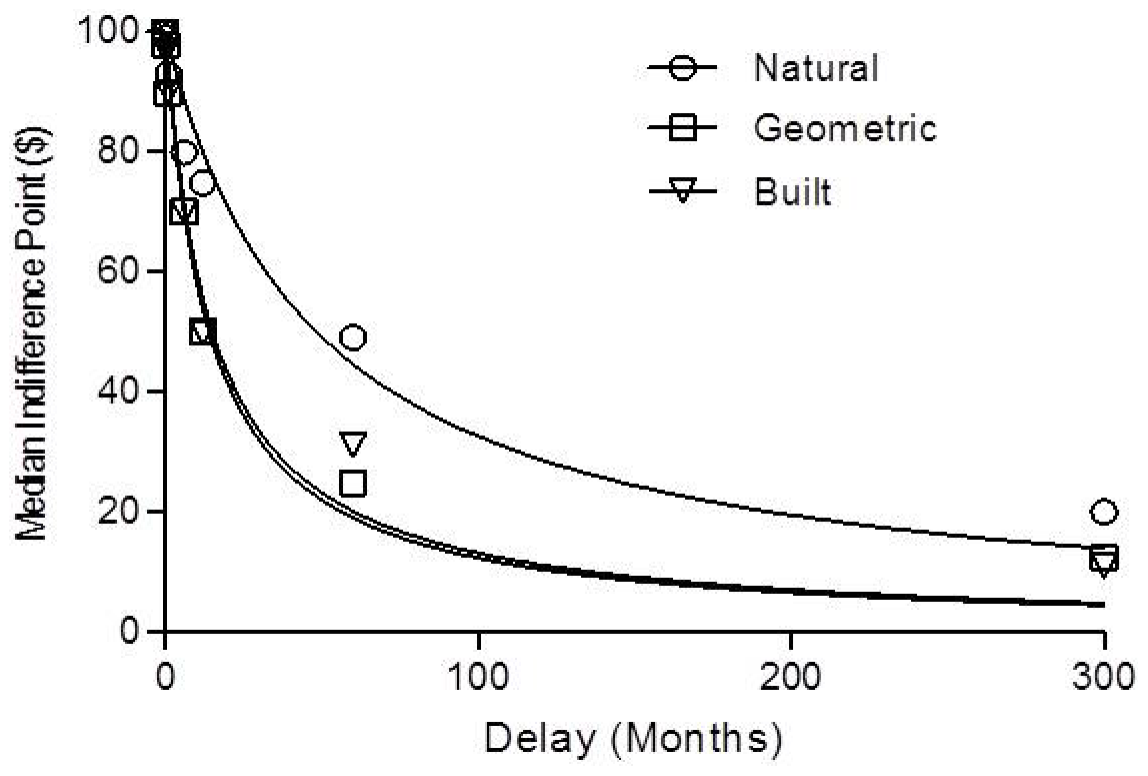
Median indifference points as a function of delay. The median indifference points as a function of delay (months) for natural (circles), geometric (squares), and built (triangles) conditions. Lines show the best fit of Equation 1 to the median indifference points.

Figure 3 shows the median indifference points decreased as delay increased in each condition; participants viewing scenes of natural environments exhibited less impulsive decision-making (higher indifference points) relative to participants who viewed built environments or geometric shapes. Equation 1 provided a good fit to median indifference points for the natural (R^2^ = .96; *k* = .02), built (R^2^ = .97; *k* = .07), and geometric conditions (R^2^ = .98; *k* = .07). Equation 1 also provided good fits to the indifference points of individual participants (Mdn R^2^ = .92,.92,.94 for natural, built, and geometric conditions). The median *k* values were.03,.07, and.06 for the natural, built and geometric conditions respectively. The Kruskal-Wallis one-way ANOVA revealed a significant difference in impulsive decision-making across conditions, F (2, 183) = 6.70, *p* = .04.

Follow up analysis confirmed that participants who viewed natural environments showed less impulsive decision-making (lower *k* values) than participants viewing built environments (Mann-Whitney t-test; U = 1405, *p* = 0.02, Cliff’s Delta = 0.24) or geometric shapes (U = 1546, *p* = 0.03, Cliff’s Delta = 0.22), with moderate effect sizes across these comparisons. There was no difference in levels of impulsive decision-making shown by participants in the geometric and built conditions (U = 1839, *p* = .92, Cliff’s Delta = 0.01). In addition to the *k* values obtained by using Equation 1, the same analyses were applied using delay discounting models proposed by Green and Myerson [55], Rachlin [56], and Takahashi [57], and yielded similar conclusions (data not shown).

## Discussion

The current study suggests that exposure to natural environments can decrease impulsive decision-making in humans. Exposure to scenes of natural environments resulted in significantly less impulsive decision-making, while viewing scenes of built environments and geometric shapes resulted in similar, higher levels of impulsive decision-making. These data converge with previous research in which similar performance on cognitive tasks was shown by viewing built environments and geometric shapes, yet improved by viewing scenes of nature [10]. Effects of natural versus built environments on mood [7], [8], attention [10], and time perception [49]–or a combination of these influences–could be driving these effects.

Mood induction influences impulsive decision-making [50], and mood is improved in natural environments [7], [8]. Positive mood induction, however, has been shown to elicit more impulsive decision-making [50]. This may be a result of augmented dopaminergic activity related to reward cues, resulting in heightened value of immediate gratification [58] while in a positive mood induced state. Therefore, if mood primarily impacted our current results, natural scenes would increase, rather than decrease, impulsive decision-making. It is important to note, however, that in a previous experiment testing the effects of mood induction on degree of delay discounting [50], the mood induction technique used was not exposure to natural scenes. It is possible that differing mood induction techniques affect degree of delay discounting differently. Enhanced mood is thus unlikely to be the driving mechanism for decreased impulsive decision-making with exposure to natural stimuli, although more research is needed to identify explicitly the effects of using natural scenes to induce mood, and the subsequent influences on degree of delay discounting.

Attention also impacts delay discounting [15], as individuals with attention deficits are more impulsive [51]. An individual with reduced attentional capacity, or whose attention is overly taxed, may have difficulty processing the consequences of complex decisions and therefore consistently opt for smaller, sooner rewards. Exposure to natural versus built environments restores attention following cognitively taxing tasks, an effect which may be influenced by differences in eye movements (e.g., saccades, fixations) associated with attentional demands of tracking scenes of natural versus built environments [48]. Although we did not deliberately tax attention in this study, viewing natural scenes could increase baseline levels of attention leaving additional attentional resources to devote to consequences of decisions. Alternatively, making decisions about money could also tax attention, subsequently resulting in greater attentional restoration by natural scenes than built scenes. Differences in attention could contribute to decreased impulsive decision-making when viewing natural scenes.

Systematic differences in time perception are also related to delay discounting, in that more impulsive individuals tend to overestimate the amount of time that has passed compared with less impulsive individuals (i.e., individuals who prefer larger, later rewards) [14]. Slowed time perception (i.e., underestimation) could have decreased impulsive decision-making in the natural condition. For example, individuals report that time seems to slow when viewing awe-eliciting scenes such as waterfalls [49]. Slowed time perception could have decreased impulsive decisions as a result of viewing scenes of nature. With slowed time perception, longer delays may be subjectively perceived as shorter (i.e., 1 year feels like 6 months). As a result, individuals viewing scenes of nature may have responded with less impulsive decision-making than those viewing built environments or geometric shapes. In the present dataset, for example, the subjective value of the reward in the nature condition at 1 year was comparable to the subjective value of the reward in the built and geometric conditions at 6 months. Future research will address this issue by performing timing tasks [14] using scenes of natural and built environments as stimuli.

Regardless of mechanism, these data are the first to show decreased impulsive decision-making when humans view natural relative to built environments or geometric shapes. This study extends previous research on the benefits bestowed by natural environments to our impulsive decision-making, highlighting the broad importance of natural settings for humans. Results provide a starting point for future research exploring the benefits of exposure to natural environments. Future studies may investigate the value of including exposure to natural environments as a treatment component for a number of maladaptive behaviors such as drug addiction and overeating. General techniques that reduce impulsive decision-making may be useful, as reducing impulsivity in one realm of decision-making has been shown to reduce impulsivity in other realms of decision-making. For example, financial management training for recovering cocaine addicts resulted in less impulsive decision-making in a delay discounting task, as well as greater abstinence rates, compared to control participants [23]. Importantly, increases in impulsive decision-making in the delay discounting task were also correlated with reduced abstinence [23]. One limitation of the present study is that only short-term exposure to scenes of natural environments was assessed. Further investigation of the long-term implications of continued exposure to natural environments is therefore needed.

Future research to better understand the benefits of exposure to natural environments on impulsive decision-making may not only help persons who suffer from disorders of impulse control, but also provide a way to improve humans’ typical decision-making. For example, real-life environmental decision-making is likely influenced by impulsivity in many manifestations [59]. Choice between small immediate environmental outcomes (improved air quality for a short time now) and large delayed environmental outcomes (improved air quality for a longer duration later) follows the same general pattern as discounting of monetary outcomes [60]. Less impulsive individuals may thus choose to take public transportation to work (and decrease overall emissions in spite of an increased delay) rather than a shorter commute in a car.

Accordingly, interventions that decrease impulsive decision-making may ultimately contribute to the preservation of natural environments, which will benefit both humans and our ecosystems. Beyond the beneficial aspects provided to humans, natural settings are also essential for ecosystem function, vital resources, wildlife habitat, and preventing continued species extinction [12], [61]–[66]. Many scientific disciplines stress the preservation of ecosystems offering biological diversity, and the present study offers additional impetus for conserving these natural environments.
